# Neighborhood Built Environment and Socioeconomic Status are Associated with Active Commuting and Sedentary Behavior, but not with Leisure-Time Physical Activity, in University Students

**DOI:** 10.3390/ijerph16173176

**Published:** 2019-08-31

**Authors:** Javier Molina-García, Cristina Menescardi, Isaac Estevan, Vladimir Martínez-Bello, Ana Queralt

**Affiliations:** 1 Department of Teaching of Musical, Visual and Corporal Expression, University of Valencia, Avda. dels Tarongers, 4, 46022 Valencia, Spain; 2 AFIPS research group, University of Valencia, 46022 Valencia, Spain; 3 COS research group, University of Valencia, 46022 Valencia, Spain; 4 Department of Nursing, University of Valencia, Jaume Roig, s/n, 46010 Valencia, Spain

**Keywords:** exercise, obesity, urban environment, walkability, active transportation, college students

## Abstract

The role of neighborhood characteristics in promoting physical activity and sedentary behaviors (SB) has not been extensively studied in university students. The study purpose was to analyze the associations of neighborhood built environment and neighborhood socioeconomic status (SES) with active commuting, leisure-time physical activity (LTPA), and SB among university students. This is a cross-sectional study of 308 undergraduate students from two urban universities in Valencia, Spain. Participants’ residential neighborhoods were classified according to walkability and SES levels. Walkability was defined as an index of three built environment attributes (i.e., residential density, land-use mix, and street connectivity) based on geographical information system data. Active commuting to and from university (ACU), active commuting in the neighborhood, LTPA, and SB were evaluated through a questionnaire. Mixed model regression analyses were performed. There were no significant SES–walkability interactions for any of the outcomes analyzed. However, university students living in more walkable areas reported two more ACU trips per week compared to those living in less walkable neighborhoods (*p* < 0.01). University students living in lower-SES neighborhoods reported more ACU trips per week than those living in higher-SES neighborhoods (*p* < 0.05). Regarding LTPA, there were no significant SES or walkability main effects. Neighborhood SES was negatively related to active commuting in the neighborhood and to time spent in SB (all *p* < 0.05). Participants living in lower-SES neighborhoods reported more active commuting per week and had the highest average minutes spent in SB. This study highlights the relevance of assessing university’s residential environment when active transportation and SB are analyzed.

## 1. Introduction

The beneficial role of physical activity on promoting population health has been extensively demonstrated [1]. Physical inactivity is a global issue that causes noncommunicable diseases such as heart disease, hypertension, diabetes or cancer and reduces life expectancy [2]. In this regard, the transition from high school to university is one of the key life transitions that is characterized by multiple changes in the daily routine and is associated with a significant decrease in physical activity levels [3,4,5]. Research indicates that regarding international recommendations (i.e., at least 30 min of moderate-to-vigorous physical activity per day in adults [1]), approximately 50% of university students are considered physically inactive [6,7]. Research also suggests that physical activity must be studied through its examination across multiple domains, because this provides deepened understanding of students’ physical activity engagement [8].

From an ecological perspective [9], there are different types of factors affecting physical activity behavior in university students. In this regard, individual and psychosocial factors have been the most analyzed in previous research [10,11,12]. Nevertheless, environmental variables and, more specifically, built environment, have been scarcely analyzed among university students. The built environment refers to human-made surroundings, from small-scale settings (e.g., houses, schools, and offices) to large-scale settings (e.g., neighborhoods, communities, and cities), as well as sidewalks, streets, and green areas [13]. Neighborhoods are one site in which the built environment might be particularly relevant for health, mainly facilitating or hindering physical activity behavior [14,15]. Neighborhood characteristics are one of the most consistent built environmental factors associated with physical activity behavior in the general adult population [9,15]. According to a recent systematic review of the effects of residential relocation on physical activity [16], one of the most consistent activity-promoting neighborhood attributes is walkability. Walkability is an estimation of how much a built environment promotes physical activity [17]. It usually includes components such as residential density, street intersection density, and land use mix [18]. However, the operationalization of the measures of walkability as well as the type or number of built environment factors considered show a large degree of variability [19,20]. For this reason, the International Physical Activity and the Environment Network (IPEN) has developed a set of protocols aimed at maximizing comparability of built environment characteristics and physical activity between different geographical contexts [15]. In the present study, the IPEN walkability index was used [18].

The studies carried out in the general adult population and young people indicate that high levels of walkability of the neighborhood are related with high levels of physical activity [21,22,23]. Walkable environments have a closer proximity to green areas and other recreational areas (e.g., sport facilities) that can facilitate leisure-time physical activity (LTPA), such as leisure-time walking [24,25]. In the case of the sedentary behaviors (SB), it is hypothesized that low-walkable neighborhoods have few opportunities for physical activity and may lead citizens to spend more time doing recreational sedentary activities (e.g., watching TV) [22]. Nevertheless, in relation to SB, findings in the literature have been inconsistent [22,23,26].

The assessment of the relationship between residential neighborhood walkability and university students’ physical activity is virtually non-existent. The limited number of studies has focused on analyzing campus walkability among US university students [27,28,29]. Recently, Horacek et al. [28] analyzed the characteristics of thirteen US university campuses and demonstrated that more walkable/bikeable environments were associated with more walking for transportation and better weight status. Rybarczyk [29] evaluated the communities surrounding a US university campus and found significant associations between residential density and intersection density with active travel to campus in a sample of faculty, staff, and students. Today, there are no studies that have focused on the analysis of the built environment of the residential neighborhoods of the students from urban universities. Urban universities are more usual in European countries and are characterized by having their schools integrated into the city urban area [30] and not in a closed geographical area, such as university campuses [27,28]. On the other hand, in addition to physical activity behavior, the relationship between neighborhood walkability and SB are unexplored among university students.

Literature has also indicated the existence of socioeconomic differences in built environments [31,32,33], suggesting that low socioeconomic status (SES) residents may be exposed to less supportive environments for active lifestyles. However, the potential role of high vs. low neighborhood SES in moderating the relationship between urban environment and physical activity or SB has not been evaluated yet in university students. According to Van Dyck et al. [34], understanding if SES variables are effect modifiers is important for several reasons. One of these reasons is related with the necessity of knowing if walkability relates similarly to health behaviors (e.g., physical activity) in high- and low-SES neighborhoods. Moreover, it is important to examine these interactions to reduce health disparities across socioeconomic groups. Literature has also shown that neighborhood SES can have a direct influence on physical activity and SB [23,26]. Nevertheless, the results are inconclusive and seem to differ according to the type of behavior and the population groups analyzed [23,34,35,36].

The present study examined the associations of neighborhood walkability and neighborhood SES with active commuting, leisure-time physical activity (LTPA), and SB among Spanish university students. In this regard, it was hypothesized that both neighborhood walkability and neighborhood SES influence university students’ active commuting, LTPA, and SB.

## 2. Materials and Methods

### 2.1. Study Design and Procedure

**Figure 1:**
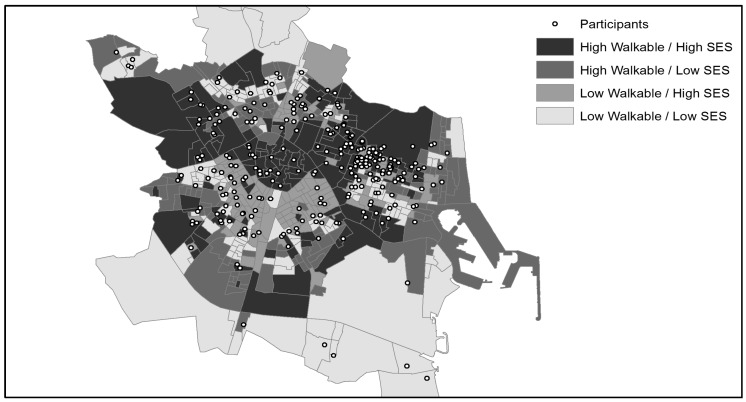
Distribution of the study participants according to the type of neighborhood in Valencia, Spain.

This study was conducted as part of the IPEN Adolescent study in Spain [23]. Although this study was designed for adolescents, the methodology was adapted to samples of children [31] and university students. A cross-sectional study was designed to recruit participants via convenience sampling in classes. Participants’ residential neighborhoods were classified according to walkability and SES levels. The smallest governmental administrative unit (i.e., census blocks) was used to delineate neighborhoods. The city of Valencia consisted of 593 census blocks at the time of the study. Census blocks were objectively evaluated and categorized as high or low walkability/SES using the IPEN walkability index [18]. Based on IPEN recommendations [18,23,37], census blocks were divided into deciles based on their walkability and SES values: The lowest five deciles constituted the “low” category, and the highest five deciles corresponded with the “high” category. Then, based on previous research [23], participants from the census blocks in the central deciles were removed. A 2 × 2 matrix was established by high/low walkability and high/low SES, with the four categories called “quadrants” (Figure 1). The use of binary variables allows comparison with other studies (e.g., [18,23,37]) from diverse types of geographical contexts.

### 2.2. Participants

Participants were 308 undergraduate students (22.4 years, standard deviation 5.6; 62.0% female) from two urban universities in Valencia (Spain) recruited via convenience sampling in classes. Previously, 11 participants were excluded because of incomplete questionnaire data. Inclusion criteria were: university students living in the city of Valencia during the academic year; and being able to walk without assistance. Data were collected in 2015 during the academic year and were balanced in the 2 × 2 matrix. Once the participants from the census blocks in the central deciles were removed, the final sample was composed of 213 university students. The study protocol was approved by the Human Research Ethics Committee at the corresponding author’s university, and informed consent was obtained from the participants before study enrollment.

### 2.3. Measures

#### 2.3.1. Neighborhood Evaluation

The IPEN walkability index score for each census block was calculated using GIS (Geographic Information System) measures of net residential density (ratio of residential units to the land area devoted to residential use), street intersection density (ratio of the number of street intersections to land area of the block-group), and land use mix (diversity of land use types per census block), as described previously (see Frank et al. [18] for more description). The formula for land use mix captures how evenly the square footage of diverse uses is distributed (i.e., residential, office, civic/institutional, recreation, entertainment, food-related, and retail area) and ranges from 0 to 1 [38]. The walkability index was the sum of the z scores of the three built environment measures:

Walkability Index = [(z-score of net residential density) + (z-score of intersection density) + (z-score of land use mix)]

The educational level of each census block was used as an indicator of SES [23,39]. These data on educational level were obtained through the INE (National Institute of Statistics, Spain) for 2011. Educational level ranged from 0 (illiterate person) to 4 (university training). The average of this variable was calculated in each census block. Then, the z score of the educational level was also calculated. ArcGIS 10.2 software (ESRI, Redlands, CA, USA) was used to generate the GIS variables.

#### 2.3.2. Active Commuting to and from University (ACU)

Modes of transport to and from university were assessed by: “How often do you use each of the following ways to go to and from the university?” [40]. Response options were bike, bus, car, train/metro/tram, motorbike, and walking. University students indicated the number of trips per week (to or from university) in each mode of transport. The total number of trips per week they made on foot or by cycling was obtained. This questionnaire has been demonstrated to be acceptably reliable and valid in university students in a previous study [40].

#### 2.3.3. Active Commuting in the Neighborhood, Leisure-Time Physical Activity (LTPA), and Sedentary Behavior (SB)

These physical activity domains were assessed by the Spanish version of the GPAQ survey (Global Physical Activity Questionnaire [41]). The GPAQ was developed and validated by the World Health Organization [41]. The information on the frequency and duration of the active transportation and moderate-and-vigorous-intensity LTPA was collected. In relation to active commuting, university students were asked to report the usual way they travel to and from places (e.g., to work, for shopping and to market). Participants were also asked to report the average sitting time per day as a proxy for SB. The GPAQ questionnaire has been satisfactorily used among Spanish university students in previous research (e.g., [5]).

#### 2.3.4. Covariates

Body mass index (BMI; kg/m^2^) was calculated using self-reported weight and height. Access to car and motorbike was also evaluated using two items [12]: “Do you have a car for personal use?”; “Do you have a motorbike for personal use?” Items were rated 1 (“never”), 2 (“sometimes”) or 3 (“always”). Type of residence was assessed by “Where do you live during the academic year?” Response options were divided into two categories: family residence (parents’ home or own house) and university residence (shared flat with other students or hall of residence). The street-network distance from participants’ residence to university school was calculated using GIS procedures. University students were also asked to indicate the number of years living at their current address. Access to public transport was also measured with: “How long does it take you to walk from your home to the nearest public transit? (bus, tram, metro).” Participants responded in minutes. Barriers to ACU were measured using a reliable and valid scale that includes items related to the environment/safety (seven items) and planning/psychosocial factors (seven items) [12]. Example items are: ‘There is nowhere to leave a bike safely’ and ‘I have too much stuff to carry to walk or bike’. This scale uses a response format from 1 (“strongly disagree”) to 4 (“strongly agree”). Other covariates were participants’ gender and age.

### 2.4. Statistical Analysis

The analyses were carried out using SPSS v.22.0 (SPSS, Chicago, IL, USA). Descriptive statistics (e.g., means, standard deviations, skewness for continuous measures, frequencies, and percentages) were calculated to analyze the distributions of the measurements.

The main variables of interest, in the models run to address the study’s primary purpose, were high- vs. low-neighborhood walkability and high- vs. low-neighborhood SES, as well as the interaction between these two variables. For each outcome variable, the full model (walkability and SES main effects, interactions, and all covariates) was first tested to determine whether there was an SES–walkability interaction effect. To minimize type 2 statistical errors, covariates with *p* > 0.15 were removed using backward-stepwise elimination procedures. Separate mixed effects regression models (using SPSS MIXED) were fit for all the dependent variables (i.e., ACU, active commuting in the neighborhood, LTPA, and SB). Mixed-regression analyses were used so that clustering of participants nested within residential neighborhoods (administrative units), and university schools could be adjusted for as random effects [23]. Sociodemographic measures tested as potential covariates were participant’s gender, age, body mass index, access to car/motorbike for personal use, type of residence, number of years living at current address, distance to university, access to public transport, and barriers to ACU.

## 3. Results

Table 1 indicates the study descriptive statistics for all the participants.

**Figure 2:**
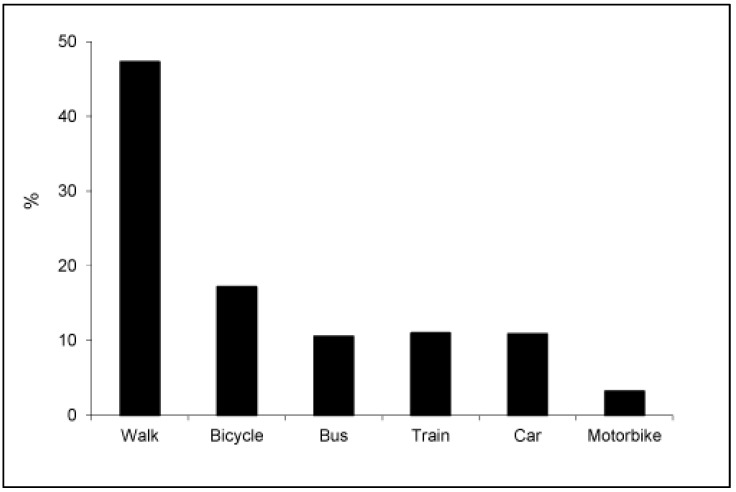
Percentages of trips to and from university by each mode of transport.

Figure 2 gives the percentage of trips to and from university by mode of transport per week. A notable percentage of students walked to and from university (47.3% of trips). The percentage of trips by bike was 17.2%, whereas 21.5% was by public transport (train and bus) and 14.0% by private motorized transport (car and motorbike).

Table 2 shows the participants’ outcomes by neighborhood-quadrant with covariate-adjusted means. The SES-by-walkability interaction (or SES and walkability main effects, if the interaction was nonsignificant) is also indicated.

Considering Table 2, there were no significant SES-by-walkability interactions for any of the outcomes analyzed. Nevertheless, ACU showed significant walkability (*p* = 0.013) and SES (*p* = 0.053) main effects. ACU was more frequent overall in higher-walkable and lower-SES areas. University students living in more walkable areas reported more ACU trips per week compared to those living in less walkable neighborhoods (9.7 vs. 7.6 trips, respectively). Likewise, university students living in lower-SES neighborhoods reported about two more trips per week in contrast to those living in higher-SES neighborhoods (9.6 vs. 7.7 trips, respectively).

In relation to active commuting in the neighborhood, there was also one neighborhood SES main effect (*p* = 0.026), with university students living in lower-SES neighborhoods reporting more active days per week than university students living in higher-SES neighborhoods (5.5 vs. 4.1 days, respectively).

Regarding LTPA, there were no significant SES or walkability main effects. However, time spent in SB showed a significant neighborhood SES main effect (*p* = 0.035). The highest average minutes spent in SB (468.6 min/day) was found among university students living in lower-SES areas in contrast to those living in higher-SES areas (average of 369.4 min).

## 4. Discussion

In a society where transition from high school to university implies an increment of SB [3,5,42] and scarce time of physical activity practice in university students [6,7], it is important to promote healthy lifestyles and contribute to increasing physical activity engagement in this population [8]. The purpose of this cross-sectional study was to examine the associations of neighborhood walkability and neighborhood SES with active commuting, LTPA, and SB among Spanish university students. The results supported that the built environment and/or neighborhood SES are associated with active commuting and SB among Spanish university students.

Transport to university is one of the four life domains contributing to physical activity levels in university students [8]. Systematic review evidence has recently found that ACU can be integrated into overall lifestyle activity to reduce obesity and promote better cardio metabolic health [43]. It should be noted that in the current study, a notable percentage of students actively commutes to and from university on foot (47.3% of trips) and by biking (17.2%). Active commuting on foot or by biking is a good strategy to incorporate physical activity into daily routines among physically inactive populations [44]. In this sense, considering the competing academic and occupational goals in university students [5], ACU might lead to them integrating physical activity engagement in daily routine easier than LTPA in other life domains (e.g., recreational or domestic) and improve healthy levels [45], such as reducing obesity [40,46,47].

Walkability of the neighborhood was a factor that influences ACU. Our main findings indicate that university students who lived in more walkable neighborhoods were more likely to actively travel to university; these students reported two trips more per week compared to those living in less walkable neighborhoods. Similar results were found in children and adolescents [23,31], reinforcing the idea of neighborhood characteristics influence ACU and, consequently, physical activity levels. The results in less walkable neighborhoods could be due to longer, isolated, and unattractive routes discouraging physical activity [44,48]. Perceived walkability is also likely to influence behavior [44,48], but this was not measured in the current study.

In addition, ACU is also associated with the neighborhood SES. In contrast to those students living in higher-SES neighborhoods, counterparts living in lower-SES neighborhoods actively commuted to/from university about twice more per week. Our study also supports previous findings, in which children and adolescents living in lower-SES neighborhoods actively commuted to school frequently [31]. This confirms the assumption that physical activity can be affected by the socioeconomic variables of the neighborhood [49]. Furthermore, there is an association between active commuting in the neighborhood and neighborhood SES. That is, students living in lower-SES areas commuted actively more in their neighborhood than those living in higher-SES neighborhoods (5.5 vs. 4.1 days/week, respectively). This could be due to students from higher-SES areas being able to afford motor vehicles (i.e., cars or motorbikes), and the possibility of access to parking facilities [31]. Considering previous research, it is clear that the use of a motor vehicle is associated with weight gain and increased risk of obesity [47]. As it seems that high-SES students from the current study are more likely to commute passively by driving to university, more active modes of transport should be promoted to favor the adoption of physical activity guidelines.

Regarding SB, an association with neighborhood SES was found. University students living in lower-SES areas spent almost 100 min/day more in SB activities compared to those living in higher-SES areas. It is suggested that among students from low-SES areas, sedentary time is spent on screen time (TV, mobile phone, laptop or computer) [50] because of the lack of sport facilities and organized physical activities promotion in their neighborhood compared to higher-SES neighborhoods [23]. In this sense, it should be noted that SB has also been related to an increase in unhealthy food consumption (e.g., sweets, savory snacks, soda, and soft drinks) and the subsequent risk of obesity [51,52]. With the emerging importance of prolonged sitting time as a chronic disease risk factor, it is important to identify the correlates of SB to develop public health interventions [53].

Regarding LTPA, in the present study, neither neighborhood SES nor walkability were associated with LTPA. These results could be due to university students participating in sports/physical activity classes [23] in different neighborhoods than those in which they live. In this sense, Spanish universities usually offer very varied sports facilities, as well as the possibility of participating in different organized sports activities [10]. Otherwise, the lack of a significant association between walkability and LTPA could be related to the limitations of habitual physical activity measurement through questionnaires [54]. The use of self-reports could be seen as a limitation of the present study because participants usually have a tendency to overreport physical activity and underreport SB [40]. As a more appropriate perspective, it would be desirable that future studies, in university students, use objective measures of physical activity behavior such as motion sensors to determine physical activity level.

### Applications for Practice

This study showed a broad group of students (i.e., 64.5%) that travel to/from university on foot, via biking or public transport. However, there is still a significant percentage of university students (14.0%) who do not commute actively. In this line, policy makers could conduct programs for physical activity promotion [55] in universities, especially those aimed at promoting active commuting. In relation to the neighborhood design, street characteristics could be designed to turn streets into a more walkable and bikeable environments. Neighborhoods’ infrastructure and aesthetics, in addition to the sense of comfort and safety, promote walkability [55,56]. On the other hand, as it seems that students from high-SES areas passively commute by driving to/from university, a strategy to encourage them to travel actively could be based on reducing the availability and parking access close to university schools or imposing high fees on parking facilities [56,57]. Moreover, as the number of students moving by car increases rapidly with increasing distance, improving accommodation facilities on or near universities (e.g., residences halls or shared flats) could also increase ACU [40]. Similarly, the improvement of public transport connection from students’ neighborhoods to university neighborhoods could provide an opportunity to promote ACU [55].

## 5. Conclusions

This study highlights the relevance of assessing a university’s residential environment when active commuting and SB are analyzed. University students living in more walkable areas reported more ACU trips per week compared to those living in less walkable neighborhoods. Moreover, ACU and active commuting in the neighborhood were more frequent in lower-SES areas than in higher-SES neighborhoods. Furthermore, time spent in SB was higher among lower-SES residents.

## Figures and Tables

**Table 1:** Study descriptives for all the sample participants.

Variables	Range	Mean (SD) or %
Sociodemographics		
Body mass index (kg/m^2^)	17.6–32.4	22.3 (2.9)
Access to car/motorbike	1–3	1.5 (0.6)
Type of residence (family)	-	56.1
No. of years living at current address	1–33	11.0 (9.4)
Distance to university (km)	0.2–9.9	2.7 (1.8)
Access to public transport (min)	0–20	4.3 (3.6)
Barriers to active commuting to university	1–4	2.4 (0.5)
Outcome variables		
Active commuting to and from university (trips/week)	0–22	8.5 (4.8)
Active commuting in the neighborhood (days/week)	0–7	4.5 (2.5)
Moderate–Vigorous LTPA (min/week)	0–2520	329.8 (387.2)
Moderate LTPA (min/week)	0–1680	154.5 (240.7)
Vigorous LTPA (min/week)	0–900	175.3 (234.2)
Sedentary behavior (min/day)	90–900	400.2 (191.1)

LTPA: leisure-time physical activity.

**Table 2:** Participants’ active transportation, leisure-time physical activity (LTPA), and sedentary behavior with adjusted means by study-design quadrants and tests for neighborhood socioeconomic status (SES)-by-walkability interaction, or the main effects of SES and walkability without interaction.

Outcome Variables	Adjusted Means (SD)				Tests of significance (*p*-Value)		
	Low Walkable		High Walkable		SES-by-Walkability Interaction (If *p* < 0.05)	SES Main Effect (If n.s. Interaction)	Walkability Main Effect (If n.s. Interaction)
	SES		SES				
	Low	High	Low	High			
Active commuting to and from university (trips/week)	8.5 (0.9)	6.6 (0.8)	10.7 (1.0)	8.8 (0.4)	0.506	**0.053**	**0.013**
Active commuting in the neighborhood (days/week)	5.4 (0.5)	4.0 (0.5)	5.7 (0.6)	4.3 (0.2)	0.957	**0.026**	0.521
Moderate–Vigorous LTPA (min/week)	354.2 (94.2)	285.2 (83.0)	415.2 (107.7)	346.2 (55.2)	0.913	0.480	0.442
Moderate LTPA (min/week)	182.5 (56.2)	94.2 (48.2)	260.6 (66.0)	172.2 (24.8)	0.591	0.175	0.139
Vigorous LTPA (min/week)	169.5 (56.7)	192.1 (49.9)	159.5 (64.6)	182.0 (33.0)	0.659	0.702	0.834
Sedentary behavior (min/day)	452.6 (47.8)	353.4 (42.3)	484.5 (52.4)	385.4 (30.8)	0.435	**0.035**	0.415

Note: Bold values indicate statistically significant differences (*p* < 0.05). Models for the main effects contain both walkability and SES factors. Abbreviations: SES, socioeconomic status; LTPA, leisure-time physical activity, n.s.: nonsignificant.
